# Di­aqua­bis­(nicotinamide-κ*N* ^1^)bis­(thio­cyanato-κ*N*)nickel(II)

**DOI:** 10.1107/S1600536814006771

**Published:** 2014-04-16

**Authors:** Deepanjali Pandey, Shahid S. Narvi, Gopal K. Mehrotra, Raymond J. Butcher

**Affiliations:** a Department of Chemistry, Motilal Nehru National Institute of Technology, Allahabad 211 004, India; b Department of Chemistry, Howard University, 2400 Sixth Street, N.W. Washington, DC 20059, USA

## Abstract

In the title complex, [Ni(NCS)_2_(C_6_H_6_N_2_O)_2_(H_2_O)_2_], the Ni^II^ ion is located on an inversion center and is coordinated in a distorted octa­hedral environment by two N atoms from two nicotinamide ligands and two water mol­ecules in the equatorial plane, and two N atoms from two thio­cyanate anions in the axial positions, all acting as monodentate ligands. In the crystal, weak N—H⋯S hydrogen bonds between the amino groups and the thio­cyanate anions form an *R*_4_^2^(8) motif. The complex mol­ecules are linked by O—H⋯O, O—H⋯S, and N—H⋯S hydrogen bonds into a three-dimensional supra­molecular structure. Weak π–π inter­actions between the pyridine rings is also found [centroid–centroid distance = 3.8578 (14) Å].

## Related literature   

For background to the applications of transition metal complexes with biochemically active ligands, see: Antolini *et al.* (1982 ▶); Krishnamachari (1974 ▶). For related structures, see: Hökelek, Dal *et al.* (2009 ▶); Hökelek, Yilmaz *et al.* (2009 ▶); Özbek *et al.* (2009 ▶); Zhu *et al.* (2006 ▶).
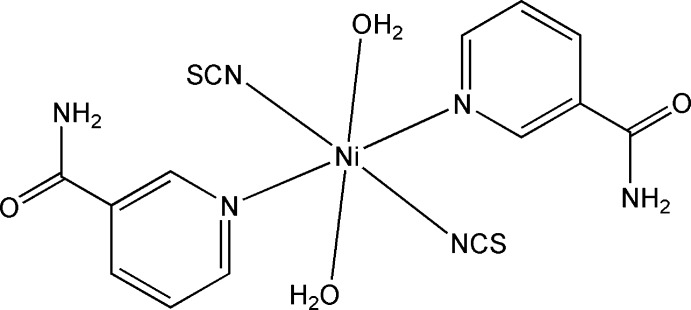


## Experimental   

### 

#### Crystal data  

[Ni(NCS)_2_(C_6_H_6_N_2_O)_2_(H_2_O)_2_]
*M* *_r_* = 455.16
Triclinic, 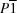

*a* = 7.5574 (15) Å
*b* = 8.2683 (19) Å
*c* = 9.0056 (15) Å
α = 73.010 (18)°
β = 69.698 (17)°
γ = 66.51 (2)°
*V* = 476.23 (18) Å^3^
*Z* = 1
Mo *K*α radiation
μ = 1.27 mm^−1^
*T* = 123 K
0.48 × 0.32 × 0.26 mm

#### Data collection  

Agilent Xcalibur Ruby CCD diffractometer
Absorption correction: multi-scan (*CrysAlis PRO*; Agilent, 2012 ▶) *T*_min_ = 0.690, *T*_max_ = 1.000
8114 measured reflections
4752 independent reflections
3477 reflections with *I* > 2σ(*I*)
*R*_int_ = 0.032

#### Refinement  

*R*[*F* ^2^ > 2σ(*F* ^2^)] = 0.046
*wR*(*F* ^2^) = 0.125
*S* = 1.03
4752 reflections
132 parameters
3 restraints
H atoms treated by a mixture of independent and constrained refinement
Δρ_max_ = 0.50 e Å^−3^
Δρ_min_ = −0.71 e Å^−3^

### 

Data collection: *CrysAlis PRO* (Agilent, 2012 ▶); cell refinement: *CrysAlis PRO*; data reduction: *CrysAlis PRO*; program(s) used to solve structure: *SHELXS97* (Sheldrick, 2008 ▶); program(s) used to refine structure: *SHELXL97* (Sheldrick, 2008 ▶); molecular graphics: *SHELXTL* (Sheldrick, 2008 ▶) and *Mercury* (Macrae *et al.*, 2008 ▶); software used to prepare material for publication: *SHELXTL*.

## Supplementary Material

Crystal structure: contains datablock(s) I. DOI: 10.1107/S1600536814006771/hy2643sup1.cif

Structure factors: contains datablock(s) I. DOI: 10.1107/S1600536814006771/hy2643Isup2.hkl

CCDC reference: 993930

Additional supporting information:  crystallographic information; 3D view; checkCIF report

## Figures and Tables

**Table 1:** Hydrogen-bond geometry (Å, °)

*D*—H⋯*A*	*D*—H	H⋯*A*	*D*⋯*A*	*D*—H⋯*A*
O1*W*—H1*W*1⋯S1^i^	0.79 (3)	2.47 (3)	3.224 (2)	161 (3)
O1*W*—H1*W*2⋯O1^ii^	0.79 (2)	1.92 (2)	2.686 (2)	164 (3)
N2—H2*A*⋯S1^iii^	0.88	2.67	3.459 (2)	150
N2—H2*B*⋯S1^iv^	0.88	2.62	3.435 (2)	154

Symmetry codes: (i) 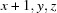; (ii) 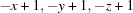; (iii) 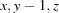; (iv) 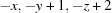.

**Fig. 1.**
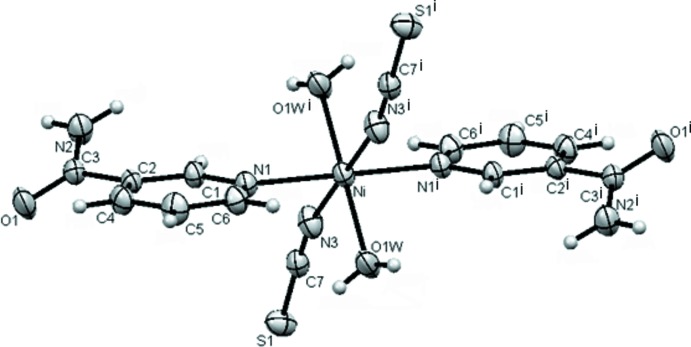
Molecular structure of the title complex, showing the 50% probability level ellipsoids. [Symmetry code: (i) 1-x, 1-y, 2-z.]

**Fig. 2.**
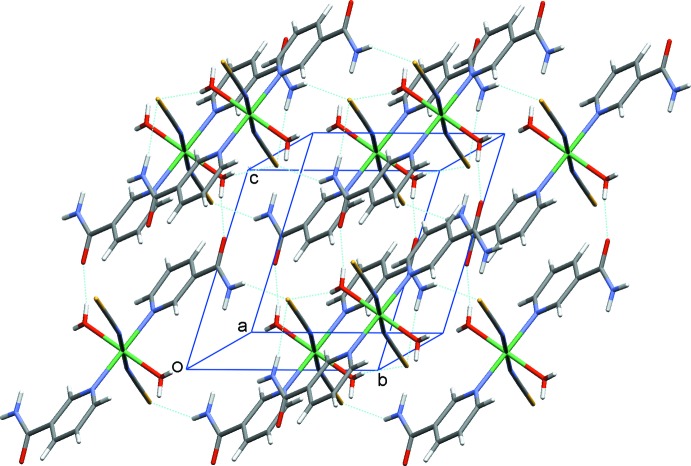
Packing diagram of the title complex. Hydrogen bonds are shown as dashed lines.

## supplementary crystallographic information

### 1. Comment

Transition metal complexes with biochemically active ligands frequently show interesting physical and/or chemical properties, as a result they may find applications in biological systems (Antolini *et al.*, 1982). As ligands, nicotinamide (NA) and thiocyanate are interesting due to their potential formation of metal coordination complexes as they exhibit multifunctional coordination modes due to the presence of S and N donor atoms. With reference to the hard and soft acids and bases concept, the soft cations show a pronounced affinity for coordination with the softer ligands, while hard cations prefer coordination with harder ligands (Hökelek, Dal *et al.*, 2009; Hökelek, Yilmaz *et al.*, 2009; Özbek *et al.*, 2009; Zhu *et al.*, 2006). NA is one form of niacin and a deficiency of this vitamin leads to loss of copper from body, known as pellagra disease. The nicotinic acid derivative *N*,*N*-diethylnicotinamide (DENA) is an important respiratory stimulant.

In the title complex, the Ni^II^ ion is located on an inversion center and coordinated by two equatorial N atoms from two NA ligands and two equatorial O atoms from water molecules, and two axial N donor from thiocyanate ligands, as can be seen in Fig. 1. The Ni—O1W bond distance is 2.088 (2) Å, which is very close to the Ni—N3(thiocyanate) distance of 2.090 (2) Å. The bond distance of Ni—N1(NA) is longer at 2.178 (1) Å. The N—Ni–N, O—Ni–N angles indicate a slightly distorted octahedral coordination for the Ni^II^ ion. The thiocyanate anion is almost linear with an N—C—S bond angle being 178.3 (2)°, coordinating in a little bent fashion to Ni with an Ni—N3—C7 angle of 160.38 (17)°. The two terminal N–bonded thiocyanate anions around the Ni^II^ ion are *trans* arranged. The Ni···Ni distance spaced by the thiocyanate ligand is 7.5574 (15) Å.

As can be seen from the packing diagram (Fig. 2), the complex molecules are linked by intermolecular O—H···O, O—H···S and N—H···S hydrogen bonds (Table 1), forming a supramolecular structure. The discrete molecules are connected by O1W—H···O1 and O1W—H···S1 hydrogen bonds into a two-dimensional layer parallel to (010). The thiocyanate S1 atom also accepts the other two hydrogen bonds from two different amide N atoms, completing an overall three-dimensional supramolecular structure.

### 2. Experimental

An aqueous solution (10 ml) of nickel acetate tetrahydrate (0.246 g, 1 mmol) and potassium thiocyanate (0.196 g, 2 mmol) was slowly added drop wise to a hot aqueous solution (10 ml) of nicotinamide (0.244 g, 2 mmol) with stirring. Greenish blue colour solution was obtained. After filtration the final clear solution left undisturbed at room temperature for slow evaporation. Next day, needle shaped greenish blue crystals were collected and dried *in vacuo* over silica gel. Crystals suitable for single crystal X-ray diffraction were manually selected and immersed in silicon oil.

### 3. Refinement

H atoms bound to C and N atoms were placed in calculated positions and refined as riding atoms, with C—H = 0.95 and N—H = 0.88 Å and with *U*_iso_(H) = 1.2*U*_eq_(C, N). H atoms of the water molecule were located from a difference Fourier map and refined isotropically.

### Crystal data

**Table:**

[Ni(NCS)_2_(C_6_H_6_N_2_O)_2_(H_2_O)_2_]	*Z* = 1
*M**_r_* = 455.16	*F*(000) = 234
Triclinic, *P*1	*D*_x_ = 1.587 Mg m^−^^3^
Hall symbol: -P 1	Mo *K*α radiation, λ = 0.71073 Å
*a* = 7.5574 (15) Å	Cell parameters from 1387 reflections
*b* = 8.2683 (19) Å	θ = 5.2–37.4°
*c* = 9.0056 (15) Å	µ = 1.27 mm^−^^1^
α = 73.010 (18)°	*T* = 123 K
β = 69.698 (17)°	Prism, green-blue
γ = 66.51 (2)°	0.48 × 0.32 × 0.26 mm
*V* = 476.23 (18) Å^3^	

### Data collection

**Table:**

Agilent Xcalibur Ruby CCD diffractometer	4752 independent reflections
Radiation source: Enhance (Mo) X-ray Source	3477 reflections with *I* > 2σ(*I*)
Graphite monochromator	*R*_int_ = 0.032
Detector resolution: 10.5081 pixels mm^-1^	θ_max_ = 37.8°, θ_min_ = 5.1°
ω scans	*h* = −12→11
Absorption correction: multi-scan (*CrysAlis PRO*; Agilent, 2012)	*k* = −13→14
*T*_min_ = 0.690, *T*_max_ = 1.000	*l* = −15→15
8114 measured reflections	

### Refinement

**Table:**

Refinement on *F*^2^	Primary atom site location: structure-invariant direct methods
Least-squares matrix: full	Secondary atom site location: difference Fourier map
*R*[*F*^2^ > 2σ(*F*^2^)] = 0.046	Hydrogen site location: inferred from neighbouring sites
*wR*(*F*^2^) = 0.125	H atoms treated by a mixture of independent and constrained refinement
*S* = 1.03	*w* = 1/[σ^2^(*F*_o_^2^) + (0.0525*P*)^2^ + 0.1045*P*] where *P* = (*F*_o_^2^ + 2*F*_c_^2^)/3
4752 reflections	(Δ/σ)_max_ < 0.001
132 parameters	Δρ_max_ = 0.50 e Å^−^^3^
3 restraints	Δρ_min_ = −0.71 e Å^−^^3^

### Special details

**Table:**

Geometry. All e.s.d.'s (except the e.s.d. in the dihedral angle between two l.s. planes) are estimated using the full covariance matrix. The cell e.s.d.'s are taken into account individually in the estimation of e.s.d.'s in distances, angles and torsion angles; correlations between e.s.d.'s in cell parameters are only used when they are defined by crystal symmetry. An approximate (isotropic) treatment of cell e.s.d.'s is used for estimating e.s.d.'s involving l.s. planes.
Refinement. Refinement of *F*^2^ against ALL reflections. The weighted *R*-factor *wR* and goodness of fit *S* are based on *F*^2^, conventional *R*-factors *R* are based on *F*, with *F* set to zero for negative *F*^2^. The threshold expression of *F*^2^ > σ(*F*^2^) is used only for calculating *R*-factors(gt) *etc*. and is not relevant to the choice of reflections for refinement. *R*-factors based on *F*^2^ are statistically about twice as large as those based on *F*, and *R*- factors based on ALL data will be even larger.

### Fractional atomic coordinates and isotropic or equivalent isotropic displacement parameters (Å^2^)

**Table:**

	*x*	*y*	*z*	*U*_iso_*/*U*_eq_	
Ni	0.5000	0.5000	1.0000	0.02711 (9)	
S1	−0.10823 (7)	0.89262 (6)	0.85626 (6)	0.03634 (11)	
O1	0.3352 (3)	0.1975 (2)	0.47112 (16)	0.0455 (4)	
O1W	0.5790 (2)	0.7206 (2)	0.85157 (16)	0.0386 (3)	
H1W1	0.671 (3)	0.737 (4)	0.859 (3)	0.061 (9)*	
H1W2	0.588 (4)	0.737 (4)	0.759 (2)	0.056 (8)*	
N3	0.2125 (2)	0.6286 (2)	0.9666 (2)	0.0371 (3)	
N1	0.5789 (2)	0.38229 (19)	0.78909 (16)	0.0268 (3)	
N2	0.2320 (3)	0.1178 (2)	0.73701 (18)	0.0365 (3)	
H2A	0.1431	0.0801	0.7281	0.044*	
H2B	0.2439	0.1106	0.8327	0.044*	
C7	0.0784 (3)	0.7361 (2)	0.92131 (19)	0.0281 (3)	
C1	0.4532 (2)	0.3212 (2)	0.76502 (18)	0.0260 (3)	
H1A	0.3295	0.3278	0.8439	0.031*	
C2	0.4940 (2)	0.2486 (2)	0.63092 (17)	0.0246 (3)	
C3	0.3476 (3)	0.1854 (2)	0.60664 (19)	0.0279 (3)	
C4	0.6734 (3)	0.2419 (3)	0.5151 (2)	0.0327 (3)	
H4A	0.7051	0.1966	0.4202	0.039*	
C5	0.8053 (3)	0.3020 (3)	0.5400 (2)	0.0363 (4)	
H5A	0.9301	0.2968	0.4631	0.044*	
C6	0.7533 (3)	0.3700 (2)	0.6787 (2)	0.0314 (3)	
H6A	0.8457	0.4095	0.6956	0.038*	

### Atomic displacement parameters (Å^2^)

**Table:**

	*U*^11^	*U*^22^	*U*^33^	*U*^12^	*U*^13^	*U*^23^
Ni	0.02907 (15)	0.03414 (16)	0.02262 (14)	−0.01311 (12)	−0.00883 (11)	−0.00525 (11)
S1	0.0317 (2)	0.0386 (2)	0.0394 (2)	−0.01499 (18)	−0.01464 (18)	0.00316 (18)
O1	0.0661 (10)	0.0612 (9)	0.0270 (6)	−0.0368 (8)	−0.0189 (6)	−0.0029 (6)
O1W	0.0531 (8)	0.0506 (8)	0.0262 (6)	−0.0332 (7)	−0.0161 (6)	0.0029 (5)
N3	0.0325 (7)	0.0469 (9)	0.0357 (8)	−0.0099 (7)	−0.0125 (6)	−0.0129 (7)
N1	0.0285 (6)	0.0331 (6)	0.0231 (5)	−0.0131 (5)	−0.0071 (5)	−0.0068 (5)
N2	0.0404 (8)	0.0497 (9)	0.0279 (7)	−0.0266 (7)	−0.0045 (6)	−0.0078 (6)
C7	0.0286 (7)	0.0367 (8)	0.0241 (6)	−0.0158 (6)	−0.0050 (6)	−0.0079 (6)
C1	0.0276 (7)	0.0333 (7)	0.0203 (6)	−0.0130 (6)	−0.0041 (5)	−0.0077 (5)
C2	0.0299 (7)	0.0275 (6)	0.0189 (6)	−0.0118 (6)	−0.0060 (5)	−0.0049 (5)
C3	0.0341 (8)	0.0289 (7)	0.0238 (6)	−0.0114 (6)	−0.0082 (6)	−0.0073 (5)
C4	0.0367 (8)	0.0396 (9)	0.0223 (6)	−0.0152 (7)	−0.0007 (6)	−0.0110 (6)
C5	0.0308 (8)	0.0472 (10)	0.0309 (8)	−0.0167 (8)	0.0017 (7)	−0.0136 (7)
C6	0.0283 (7)	0.0384 (8)	0.0303 (7)	−0.0131 (7)	−0.0073 (6)	−0.0077 (7)

### Geometric parameters (Å, º)

**Table:**

Ni—O1W	2.0876 (15)	N2—H2A	0.8800
Ni—N3	2.0899 (17)	N2—H2B	0.8800
Ni—N1	2.1776 (14)	C1—C2	1.389 (2)
S1—C7	1.6377 (18)	C1—H1A	0.9500
O1—C3	1.228 (2)	C2—C4	1.386 (2)
O1W—H1W1	0.79 (2)	C2—C3	1.497 (2)
O1W—H1W2	0.79 (2)	C4—C5	1.380 (3)
N3—C7	1.158 (2)	C4—H4A	0.9500
N1—C6	1.334 (2)	C5—C6	1.387 (2)
N1—C1	1.340 (2)	C5—H5A	0.9500
N2—C3	1.322 (2)	C6—H6A	0.9500
			
O1W^i^—Ni—O1W	180.00 (6)	C3—N2—H2A	120.0
O1W^i^—Ni—N3^i^	88.85 (7)	C3—N2—H2B	120.0
O1W—Ni—N3^i^	91.15 (7)	H2A—N2—H2B	120.0
O1W^i^—Ni—N3	91.15 (7)	N3—C7—S1	178.30 (17)
O1W—Ni—N3	88.85 (7)	N1—C1—C2	123.52 (15)
N3^i^—Ni—N3	180.0	N1—C1—H1A	118.2
O1W^i^—Ni—N1	90.25 (6)	C2—C1—H1A	118.2
O1W—Ni—N1	89.75 (6)	C4—C2—C1	117.97 (16)
N3^i^—Ni—N1	92.52 (6)	C4—C2—C3	120.08 (14)
N3—Ni—N1	87.48 (6)	C1—C2—C3	121.91 (15)
O1W^i^—Ni—N1^i^	89.75 (6)	O1—C3—N2	121.81 (17)
O1W—Ni—N1^i^	90.25 (6)	O1—C3—C2	121.07 (16)
N3^i^—Ni—N1^i^	87.48 (6)	N2—C3—C2	117.12 (14)
N3—Ni—N1^i^	92.52 (6)	C5—C4—C2	118.94 (15)
N1—Ni—N1^i^	180.000 (1)	C5—C4—H4A	120.5
Ni—O1W—H1W1	118 (2)	C2—C4—H4A	120.5
Ni—O1W—H1W2	119 (2)	C4—C5—C6	119.16 (17)
H1W1—O1W—H1W2	107 (2)	C4—C5—H5A	120.4
C7—N3—Ni	160.38 (17)	C6—C5—H5A	120.4
C6—N1—C1	117.68 (14)	N1—C6—C5	122.70 (17)
C6—N1—Ni	121.18 (12)	N1—C6—H6A	118.7
C1—N1—Ni	121.14 (11)	C5—C6—H6A	118.7
			
O1W^i^—Ni—N3—C7	−179.4 (4)	Ni—N1—C1—C2	178.49 (12)
O1W—Ni—N3—C7	0.6 (4)	N1—C1—C2—C4	−1.0 (2)
N1—Ni—N3—C7	−89.2 (4)	N1—C1—C2—C3	−178.64 (14)
N1^i^—Ni—N3—C7	90.8 (4)	C4—C2—C3—O1	−30.6 (2)
O1W^i^—Ni—N1—C6	−130.61 (14)	C1—C2—C3—O1	147.02 (17)
O1W—Ni—N1—C6	49.39 (14)	C4—C2—C3—N2	149.97 (17)
N3^i^—Ni—N1—C6	−41.75 (14)	C1—C2—C3—N2	−32.4 (2)
N3—Ni—N1—C6	138.25 (14)	C1—C2—C4—C5	1.9 (3)
O1W^i^—Ni—N1—C1	50.01 (13)	C3—C2—C4—C5	179.63 (16)
O1W—Ni—N1—C1	−129.99 (13)	C2—C4—C5—C6	−1.0 (3)
N3^i^—Ni—N1—C1	138.87 (13)	C1—N1—C6—C5	1.9 (3)
N3—Ni—N1—C1	−41.13 (13)	Ni—N1—C6—C5	−177.55 (14)
C6—N1—C1—C2	−0.9 (2)	C4—C5—C6—N1	−0.9 (3)

Symmetry code: (i) −*x*+1, −*y*+1, −*z*+2.

### Hydrogen-bond geometry (Å, º)

**Table:**

*D*—H···*A*	*D*—H	H···*A*	*D*···*A*	*D*—H···*A*
O1*W*—H1*W*1···S1^ii^	0.79 (3)	2.47 (3)	3.224 (2)	161 (3)
O1*W*—H1*W*2···O1^iii^	0.79 (2)	1.92 (2)	2.686 (2)	164 (3)
N2—H2*A*···S1^iv^	0.88	2.67	3.459 (2)	150
N2—H2*B*···S1^v^	0.88	2.62	3.435 (2)	154

Symmetry codes: (ii) *x*+1, *y*, *z*; (iii) −*x*+1, −*y*+1, −*z*+1; (iv) *x*, *y*−1, *z*; (v) −*x*, −*y*+1, −*z*+2.
